# Relationship between the timing of the first postpartum ovulation and antral follicle counts in Holstein cows

**DOI:** 10.1186/s13048-020-0610-5

**Published:** 2020-01-11

**Authors:** Eri Furukawa, Tomoyuki Masaki, Kenichiro Sakaguchi, Min Bo, Yojiro Yanagawa, Koichiro Ueda, Masashi Nagano

**Affiliations:** 1 Laboratory of Theriogenology, Graduate School of Veterinary Medicine, Hokkaido University, Sapporo, 060-0818 Japan; 2 Laboratory of Animal Production System, Graduate School of Agriculture, Hokkaido University, Sapporo, 060-8589 Japan; 3 Laboratory of Theriogenology, Faculty of Veterinary Medicine, Hokkaido University, Sapporo, 060-0818 Japan; 4 Laboratory of Animal Production System, Research Faculty of Agriculture, Hokkaido University, Sapporo, 060-8589 Japan; 5 Laboratory of Animal Reproduction, Department of Animal Science, School of Veterinary Medicine, Kitasato University, Towada, 034-8628 Japan

**Keywords:** Antral follicle count, First postpartum ovulation, Ovarian reserve, Ultrasonography

## Abstract

**Background:**

The timing of the first postpartum ovulation is an important factor affecting the timing of estrous resumption in dairy cows. The first postpartum ovulation is delayed in cows producing large amounts of milk with an intensive negative energy balance. The antral follicle count (AFC) and serum anti-Müllerian hormone concentrations are known to be indicators of the ovarian reserve, which is the number and quality of follicles left in a pair of ovaries and known as an indicator of female fertility. Cows with higher AFC have been proven to show higher pregnancy rate and shorter calving to conception intervals; however, the relationship between the timing of the first postpartum ovulation and ovarian reserve remains unclear. Therefore, this study examined the relationships between postpartum follicular dynamics, the ovarian cycle, nutritional status, and ovarian reserve.

**Methods:**

Transrectal ultrasonography was conducted from calving to 70–120 days in milk (DIM) in 26 cows to monitor AFC, follicular dynamics and the ovarian cycle. Body weight (BW) and milk yield were used as indicators of nutritional status.

**Results:**

The first postpartum ovulation was significantly later in cows with low AFC (< 25) than in those with high AFC (≥25), while changes in BW from calving to the nadir and milk production were similar in both groups. The present results also suggested that cows with low AFC and a delayed first postpartum ovulation had a shorter first ovarian cycle after the first postpartum ovulation. The mean DIM of the first postpartum artificial insemination (AI) and days open (days from calving to AI with which pregnancy was achieved) were similar in high and low AFC groups.

**Conclusions:**

The first postpartum ovulation was significantly earlier in cows with high AFC than in those with low AFC. The assumed reason for this result was higher sensitivity to luteinizing hormone and larger androstenedione and estradiol production in follicles in high AFC cows. Therefore, cows with high AFC may be more fertile than those with low AFC while their milk production increase and BW decrease; it means they are in negative energy balance.

(340/350 words)

## Background

In cattle, a surge in the secretion of follicle-stimulating hormone (FSH) occurs 4–5 days after parturition [1], and the first follicular wave then emerges. A dominant follicle of the first follicular wave results in ovulation, regression, or cyst formation [2]. If the first dominant follicle ovulates, an ovarian cycle accompanied with corpus luteum (CL) formation resumes. In cases in which the first dominant follicle degenerates, second and more follicular waves repeat emergence, growth, and regression until the first ovulation occurs [2]. Since there is no CL or progesterone secretion at the time of the first ovulation, most cows do not exhibit estrous symptoms and only approximately 10% of cows show standing estrus [3]. However, in the second ovulation after the first ovarian cycle, which is defined as the period from the first to second postpartum ovulation accompanied by CL formation [4], the proportion of cows exhibiting estrous symptoms increases to 40% [3]. These findings indicate that the opportunity for first artificial insemination (AI) based on estrus detection is delayed when the time of the first postpartum ovulation becomes later in days in milk (DIM). Therefore, the resumption of an ovarian cycle is important for reproductive management in cattle.

In dairy cows, the main factor affecting the resumption of an ovarian cycle is the energy balance [5, 6]. Cows with a negative energy balance (NEB) show a suppressed frequency of gonadotropin-releasing hormone (GnRH) pulses, a shortage of luteinizing hormone (LH) pulse frequencies, and lower estradiol production, which lead to anovulatory dominant follicles [7]. In contrast, the dominant follicle in cows with a smaller NEB may ovulate earlier postpartum. The energy intake and milk yield interaction affects the energy balance in cows, and changes in the body condition score (BCS) and body weight (BW) reflect changes in nutritional status. Previous studies reported that the first postpartum ovulation was delayed in cows with acute BCS loss [5] and greater BW loss [8]. Since BW is not affected by differences in the technician evaluations and postpartum BW loss is not affected by parity [9], BW may be a more objective parameter than BCS. Advances in genetics and management in dairy farms have progressed rapidly in the last few decades and milk yield has recently increased; therefore, NEB in postpartum cows is becoming larger. More cows showed delayed first ovulation in 1998 than in 1986 because of the higher proportion of NEB cattle [10]. The proportion of cows remaining anovular during the voluntary waiting period (VWP, 50–65 DIM) was reported to be 5–40% [11–13].

The ovarian reserve, which is defined as the number and quality of follicles left in a pair of ovaries at any given time [14], is an indicator of female fertility in mono-ovulatory species, such as humans [14] and cattle [15]. Although it is not possible to directly measure the primordial follicle pool in the ovaries, the antral follicle count (AFC), which is the number of antral follicles in a pair of ovaries counted by ultrasonography, and the plasma concentration of anti-Müllerian hormone (AMH) are indicators of the ovarian reserve. AFC has been shown to positively correlate with the number of primordial follicles in the ovaries [16]. AMH is a hormone that functions to suppress the outgrowth of primordial follicles [17] and its expression has been observed in the granulosa cells of growing preantral and antral follicles [18]. Plasma AMH concentrations positively correlated with the number of primordial follicles and AFC in humans [19] and cattle [15]. Although the ovarian reserve is proven to decrease as one’s age progresses in humans [20], there is no report about negative correlation between the age and AFC in dairy cattle. Previous studies indicated high repeatability in the mean AFC values per follicular wave within an individual [21] and high repeatability in the maximum AFC value during successive follicular waves in an estrous cycle [22]. Also, this repeatability was confirmed to continue at least 1 year [22].

Previous studies demonstrated that cows with high AFC had higher pregnancy rates [23] and shorter calving to conception intervals [23, 24]; in addition, Mossa et al. [23] indicated higher parities in high AFC cows than in low AFC cows. These findings suggest better reproductive competence and the possibility of earlier first postpartum ovulation in high AFC cattle; however, the relationship between the postpartum resumption of an ovarian cycle and the ovarian reserve currently remains unclear.

The objective of the present study was to examine the relationships between follicular dynamics and the ovarian cycle, nutritional status, and ovarian reserve (AFC or serum AMH concentration) in postpartum cows.

## Methods

### Animals

Twenty-six Holstein dairy cows were used in the present study. Their parity was 3.0 ± 1.6 (mean ± SD; ranging from 1 to 6). They were kept in the Experimental Farm of Hokkaido University (Sapporo, Japan), which has tie stall barns and is attached to a small pasture, and were milked twice daily (9:00 and 15:30). The mean 305-day milk yield of these cows was 6419 kg. Each cow was subjected to the experiment from calving to 70–120 DIM. Eighteen out of 26 cows were subjected to the experiment between November 2010 and October 2011 (5 primiparous cows and 13 multiparous cows). They were housed in the barn all day between November 2010 and March 2011 and fed hay, corn silage, and concentrated feed (beet pulp and barley, 6–8 kg). Between April and October 2011, they were pastured in the daytime and nighttime and fed corn silage and a reduced amount of concentrated feed (1–3.5 kg). The 8 other cows were used between February 2013 and December 2013 (2 primiparous cows and 6 multiparous cows). They were housed in the barn all day from calving to 60 DIM. They were fed corn silage and hay at 110% the amount they had the day before as roughage, and were also fed formula feed (Monster 21, Hokuren Agricultural Cooperative Association, Sapporo, Japan) 4 kg/day, soybean meal 2 kg/day, and rolled dry corn 4 kg/day as concentrated feed. After 61 DIM, they went under either condition: pastured daytime and nighttime or housed in the barn all day. In barn housing, cows were fed corn silage, hay, and formula feed.

VWP was set to be approximately 60 DIM, and cows were inseminated when they exhibited estrous symptoms or ovulation was considered to be upcoming based on transrectal ultrasonography after VWP.

This experiment was implemented according to the animal experimental regulations of Hokkaido University (Approval nos.: 11–0013 and 12–0027).

### Ovarian ultrasonography

Transrectal ultrasonography of the ovaries was conducted by two technicians using two ultrasonographic devices, SSD-900 (ALOKA, Tokyo, Japan) with a 7.5-MHz convex probe (UST-995-7.5, ALOKA, Tokyo, Japan) and HS-1500 (HONDA ELECTRONICS CO., LTD., Aichi, Japan) with a 10.0-MHz linear probe (HLS-375MR, HONDA ELECTRONICS CO., LTD., Aichi, Japan). Scanning was conducted from 5 to 10 DIM to 70–120 DIM thrice a week (Monday, Wednesday, and Friday) in principal.

Examinations of AFC, follicular diameters, and CL formation were conducted according to procedures described by Jaiswal et al. [25]: *i.e*., depicted images of follicles (larger than approximately 2 mm in diameter) and CL in an ovary were sketched from the outside to inside of the ovary, and the diameters and numbers of follicles were then recorded. In the analysis of recorded ovarian images, a certain series of follicular dynamics as small follicles (2–5 mm) emerge, grow, are selected one by one, and reach 8.5 mm in diameter, and 1 or 2 dominant follicle(s) keep growing and result in ovulation or regression, which is defined as a follicular wave. According to previous studies [26, 27], the growth rate of follicles was calculated as 1.9 mm/day and the day of emergence of the follicular wave was defined as the first day that a follicle attained 4 mm in diameter [22, 27]. The ovulation day was selected as an intermediate day before and after the disappearance of a dominant follicle in ultrasonographic examinations. When the ultrasonographic examination was performed on 2 consecutive days and the disappearance of the dominant follicle was confirmed, the latter day was denoted as the ovulation day. Examinations of AFC were repeated from 14 to 51 times for each cow and the mean and the median AFC values in each cow was calculated.

When the largest follicle exceeded 25 mm in diameter and persisted for more than 10 days in the absence of CL, it was classified as a follicular cyst [28]. The data of 1 cow with a follicular cyst before the first postpartum ovulation were excluded from all analyses.

Since ovulation was not confirmed in 2 cows, the ovulation day was defined as the middle day (20 and 92 DIM) between the last observation before ovulation (15 and 86 DIM) and the first observation after CL formation (24 and 97 DIM, respectively), and their data were used in analyses.

### BW measurements

BW was measured using an electronic body weight scale in a frequency from once a week to once a month. According to the methods described by Roche et al. [29], BW measured within 1 week of calving was defined as BW at calving. The BW change rate from calving to the nadir ((BW at nadir - BW at calving) / BW at calving) (%), and the daily rate of BW changes from calving to the nadir (kg/day) were calculated. The data of 2 cows for which BW at calving was not measured were excluded from the analysis related to BW. The data of 6 more cows that showed nadir BW at calving were excluded from the analysis of the daily rate of BW changes from calving to the nadir.

### Blood sampling and measurement of serum AMH concentrations

The collection of blood samples and measurement of serum AMH concentrations were conducted on 18 out of 26 cows. The collection of blood samples via the caudal vein was started within 30 DIM and conducted once a month (3–4 times in 16 cows, twice in 1 cow, and once in 1 cow). The mean value of serum AMH concentrations in each cow was calculated and used in analyses. Collected samples were centrifuged at 10,000×*g* for 25 min and serum was then stored at − 20 °C until assayed for AMH.

Serum AMH concentrations were measured using AMH Gen II ELISA (A73818, BECKMAN COULTER, CA, US) and AMH Gen II Calibrators & Control (A73819, BECKMEN COULTER). Assays were conducted in accordance with the attached instructions. This assay kit can be applied for measuring AMH in cattle as described elsewhere [30].

### Statistical analysis

**Fig. 1:**
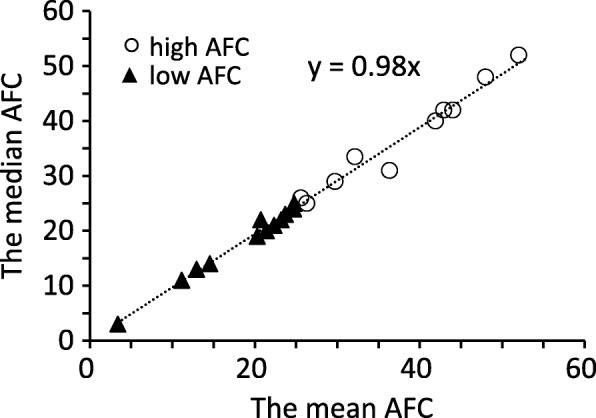
Relationship between the mean and median AFC in cows (*r* = 0.99, *P* < 0.0001). The mean and median AFC values were calculated using more than 14 samples in each cow during the experimental period

The data of 2 cows that did not ovulate in the experimental period were excluded from all analyses. The mean AFC value of each cow was used in the analyses as a representative AFC value of each cow, because they showed the positive correlation between the mean and the median AFC values in each cow (*r* = 0.99, *P* < 0.0001) (Fig. 1). Based on the results of the mean AFC value, cows were divided into two groups according to Ireland et al. [16] with slight modifications. Although cattle were divided into three groups in the study by Ireland et al. [16] based on AFC: high (≥25), intermediate (16–24), and low (≤15) groups, cows were divided into two groups in the present study: high (≥25) and low (< 25) groups. One cow in the low AFC group that had its first postpartum ovulation 87 DIM after the administration of a GnRH analog (Conceral® injection, Intervet Co., Tokyo, Japan) was included in the analysis of the first ovulation. DIM of the first AI and days open (days from calving to AI with which pregnancy was achieved) were examined in a retrospective analysis. The data of 2 cows that were not inseminated were excluded from the analysis on DIM of the first postpartum AI, and the data of 10 cows that did not conceive by the determined cut-off date, 180 DIM [4], were excluded from the analysis of days open. The data of 1 cow that did not achieve the second ovulation in the experimental period and another 2 cows in which the exact timing of ovulation was not confirmed were excluded from the analysis on the first ovarian cycle. The average milk yield (kg/day) for 17 weeks after parturition and the peak milk yield (kg/day) were analyzed according to the method of Westwood et al. [31].

All statistical analyses were performed using JMP Pro 14.0.0 (SAS Institute Japan, Tokyo, Japan). Data were presented as means ± SD, and also medians were shown in the data of AFC, AMH, DIM of the first postpartum ovulation, DIM of the first AI and days open. Mean values between 2 groups were compared using the Student’s *t*-test, the relationships between 2 parameters were analyzed using Pearson’s correlation coefficient, and *P*-values were calculated by a regression analysis.

## Results

**Fig. 2:**
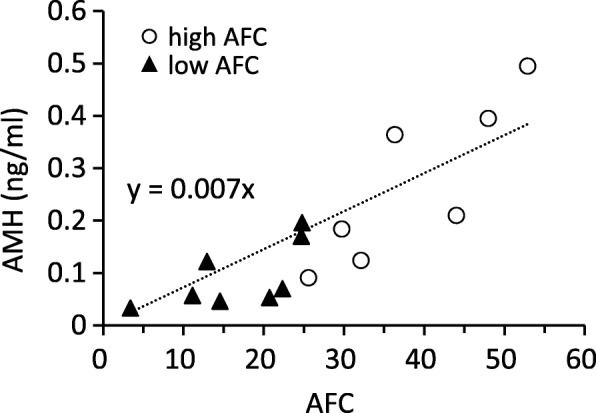
Relationship between AFC and serum AMH concentrations in cows (*r* = 0.87, *P* < 0.0001). AFC and AMH values are mean values calculated using more than 14 and 1 sample(s), respectively, in each cow during the experimental period

The average AFC of all cows was 27.2 ± 12.2 (mean ± SD, range between 3.4 and 52.0, *n* = 23); AFC was significantly higher in the high AFC group than in the low AFC group (*P* < 0.0001) (Table 1). The average AFC of 2 cows that did not ovulate in the experimental period were 14.4 and 18.6, respectively. The parity of the cows was significantly higher in the high AFC group (3.7 ± 1.5, 1 primiparous and 9 multiparous cows) than in the low AFC group (2.4 ± 1.5, 5 primiparous and 8 multiparous cows) (*P* < 0.05), and the mean months of age were higher in the high AFC group (70.3 ± 25.8) than in the low AFC group (47.3 ± 21.3) (*P* < 0.05). The average AFC were similar between cows examined by 2 different ultrasonographic devices, 26.7 ± 13.9 (SSD-900) and 28.0 ± 9.1 (HS-1500) (*P* = 0.81). The average serum AMH concentration in all cows was 0.17 ± 0.14 ng/ml (range between 0.03 and 0.50, *n* = 15); AMH was higher in the high AFC group than in the low AFC group (*P* < 0.05) (Table 1). The average AMH concentrations of 2 cows that did not ovulate in the experimental period were 0.12 and 0.11 ng/ml, respectively. Variations larger than 10-fold were noted in AFC and AMH between individuals. A strong positive correlation was observed between average AFC and serum AMH concentrations in an individual (*r* = 0.87, *P* < 0.0001) (Fig. 2). Diseases during the experimental period were hypocalcemia in 2 cows, retention of placenta in 3 cows, and mastitis in 3 cows; however, all of them were not severe and no negative effect on AFC was recognized before and after diseases.

**Table 1:** AFC and AMH of cows in the high and low AFC groups

Item	AFC group	No. of cows	Mean ± SD	Min	Median	Max
AFC	High	10	37.8 ± 9.3^a^	25.6	39.1	52.0
	Low	13	19.0 ± 6.5^b^	3.4	21.1	24.8
AMH^†^ (ng/ml)	High	7	0.27 ± 0.15^a^	0.09	0.21	0.50
	Low	8	0.09 ± 0.06^b^	0.03	0.06	0.20

*AFC* antral follicle count; *AMH* anti-Müllerian hormone. The mean AFC value of each cow was used as a representative AFC value of each cow

^ab^Values with different superscripts are significantly different (*P* < 0.05)

^†^Serum AMH concentrations were not measured in 8 cows

**Fig. 3:**
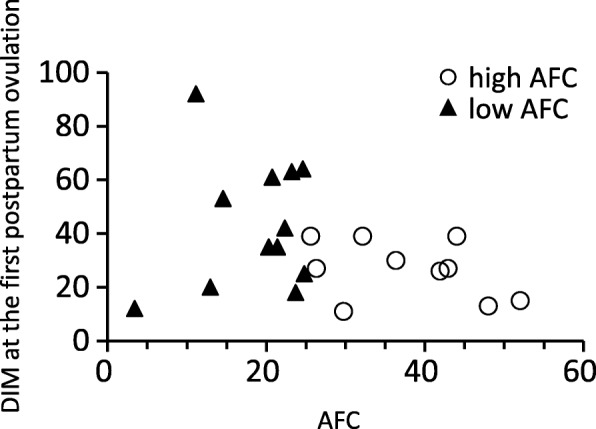
Relationship between AFC and DIM at the first postpartum ovulation. A significant difference was observed in the mean DIM of the first postpartum ovulation between the high (26.6 ± 10.7 DIM, *n* = 10; open circle symbols) and low (46.7 ± 25.8 DIM, *n* = 13; solid triangle symbols) AFC groups (*P* < 0.05). AFC values were calculated using more than 14 samples in each cow during the experimental period

The mean DIM of the first postpartum ovulation was 38.0 ± 22.7 (range between 11 and 92, *n* = 23). The first postpartum ovulation was significantly earlier in the high AFC group than in the low AFC group (*P* < 0.05) (Table 2, Fig. 3). The mean DIM of the first postpartum AI was 100.4 ± 29.5 (range between 55 and 160, *n* = 21), and were similar between the high and low AFC groups (*P* = 0.48). Furthermore, the mean days open was 113.2 ± 34.7 (range between 71 and 160, *n* = 13), and were similar between the high and low AFC groups (*P* = 0.08) (Table 2). In the low AFC group, all primiparous cows (5 out of 5) and 2 out of 7 multiparous cows (28.6%) conceived before 147 DIM. On the other hand, 6 out of 9 (66.7%) multiparous cows conceived before 160 DIM in the high AFC group.

**Table 2:** Reproductive results of cows in the high and low AFC groups

Days from parturition to	AFC group	No. of cows	Mean ± SD	Median
First postpartum ovulation	High	10	26.6 ± 10.7^a^	27
	Low	13	46.7 ± 25.8^b^	42
First postpartum AI^†^	High	9	105.8 ± 37.2	95
	Low	12	96.3 ± 23.1	93
Achieving pregnancy^‡^	High	6	131.2 ± 29.2	135
	Low	7	97.7 ± 33.0	82

*AFC* antral follicle count; *DIM* days in milk; *AI* artificial insemination

^ab^Values with different superscripts are significantly different (*P* < 0.05)

^†^Two cows were not inseminated

^‡^Days of achieving pregnancy were calculated based on cows conceived by 180 DIM

**Fig. 4:**
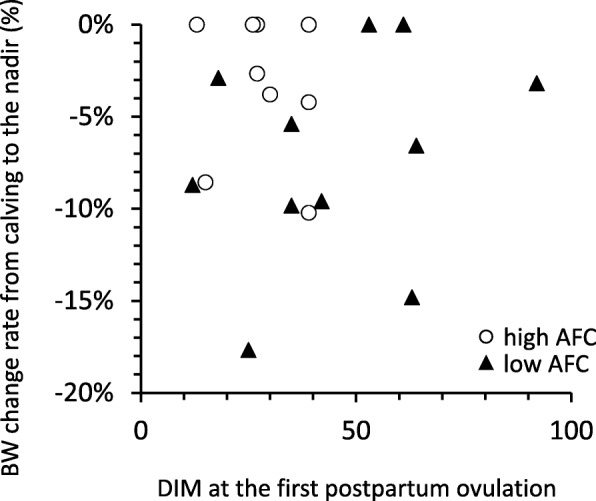
DIM at the first postpartum ovulation and BW change rate from calving to the nadir

The mean BW change rate (%) from calving to the nadir in all cows was − 5.2 ± 5.2% (range between − 17.7 and 0%, *n* = 21), and the mean weeks after parturition in which their BW became nadir was 5.6 ± 4.7 weeks (range between 1 and 16 weeks). The mean BW change rates were similar between the high and low AFC groups (*P* = 0.15) (Table 3), and also similar between primiparous (− 8.0 ± 5.0%, *n* = 5) and multiparous cows (− 4.3 ± 5.1%, *n* = 16) (*P* = 0.18). DIM of the first ovulation markedly varied in the low AFC group (from 12 to 92 DIM) regardless of BW change rate (*r* = 0.39, *P* = 0.21, *n* = 12), whereas variations in DIM of the first ovulation were smaller and first ovulation occurred earlier (range between 13 and 39 DIM) in the high AFC group regardless of BW change rate (*r* = − 0.14, *P* = 0.72, *n* = 9) (Fig. 4). The mean daily rate of BW changes from calving to the nadir in all cows was − 1.5 ± 1.3 kg/day (*n* = 15) and the mean daily rates of BW changes were similar between the high and low AFC groups (*P* = 0.74). The average milk yield for 17 weeks postpartum in all cows was 26.3 ± 5.8 kg/day (*n* = 23) and similar between the high and low AFC groups (*P* = 0.95) (Table 3). The mean peak milk yield in all cows was 32.2 ± 6.3 kg/day (*n* = 23). The mean week after parturition in which their milk production achieved their peaks was 6.6 ± 4.0 weeks (range between 2 and 17 weeks). The mean peak milk yields were similar between the high and low AFC groups (*P* = 0.89) (Table 3).

**Table 3:** Parameters of nutritional status of cows in the high and low AFC groups

Item	AFC group	No. of cows	Mean ± SD
BW change rate^ac^ (%)	High	9	−3.3 ± 3.9
	Low	12	−6.6 ± 5.7
Daily rate of BW changes^bd^ (kg/day)	High	5	−1.4 ± 0.9
	Low	10	−1.6 ± 1.5
The average milk yield^e^ (kg/day)	High	10	26.4 ± 5.7
	Low	13	26.2 ± 6.2
The mean peak milk yield^f^ (kg/day)	High	10	32.0 ± 5.4
	Low	13	32.4 ± 7.2

*AFC* antral follicle count; *BW* body weight

^a^ Two cows were not measured their BW at calving

^b^ Six cows showed nadir BW at calving

^c^ The BW change rate from calving to the nadir; ((BW at nadir - BW at calving) / BW at calving)

^d^ Daily rate of BW changes from calving to the nadir

^e^ The average milk yield was calculated by the milk yield for 17 weeks postpartum

^f^ The peak milk yield was observed from 2 to 17 weeks postpartum

**Fig. 5:**
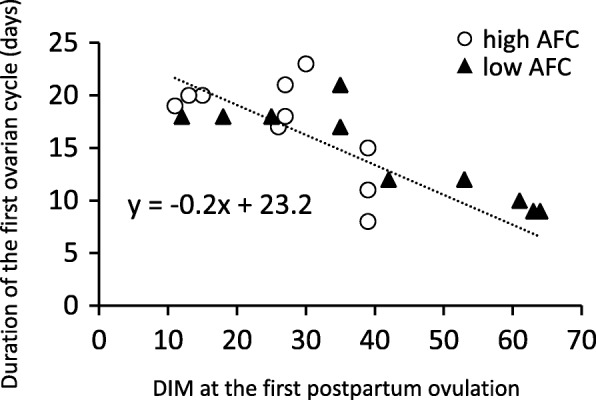
Relationship between DIM at the first postpartum ovulation and duration of the first ovarian cycle (*r* = − 0.78, *P* < 0.0001)

The mean duration of the first ovarian cycle in all cows was 15.8 ± 4.7 days (*n* = 20). Seven cows (2 cows in the high AFC group and 5 cows in the low AFC group) had a first ovarian cycle that was shorter than 12 days, with 6 (1 cow in the high AFC group and 5 cows in the low AFC group) showing only one follicular wave in the first ovarian cycle. Twelve out of 12 cows with the first postpartum ovulation within 35 DIM had a first ovarian cycle that was longer than 17 days, and 7 out of 8 cows with the first ovulation after 39 DIM had a first ovarian cycle that was shorter than 12 days (Fig. 5). A strong negative correlation was observed between DIM of the first postpartum ovulation and the duration of the first ovarian cycle (*r* = − 0.78, *P* < 0.0001). The mean duration of the first ovarian cycle was similar between the high AFC group (17.2 ± 4.7 days, *n* = 10) and low AFC group (14.4 ± 4.5 days, *n* = 10) (*P* = 0.19); however, all cows with the first ovulation after 42 DIM belonged to the low AFC group, and had a first ovarian cycle that was shorter than 12 days (Fig. 5).

## Discussion

The first postpartum ovulation was significantly earlier in high AFC cows than in low AFC cows in the present study. Mossa et al. [23] previously reported that cows with low AFC (≤15) have lower pregnancy rates and longer calving to conception intervals. The present results support these findings. Cows with low AFC are at a higher risk of delayed first postpartum ovulation, resulting in a greater risk of the delayed resumption of estrous symptoms and AI.

Changes in BW and milk production were used as indicators of nutritional status in the present study, and the interactions between the ovarian reserve and nutritional status to postpartum follicular dynamics and the ovarian cycle were investigated. Cows in different AFC groups showed a similar nutritional status; however, they showed different timing for the first postpartum ovulation; *i.e*., the timing of the first postpartum ovulation was early (from 13 to 39 DIM) in high AFC cows and largely varied (from 12 to 92 DIM) in low AFC cows. In the present study, BW decreased but milk production increased in each group. It means that they were in undernutrition condition and assumed to be in NEB status. These results suggest the ovarian reserve is an important regulatory factor controlling the timing of the first ovulation under NEB status. In further study, we should evaluate dry matter intake and metabolic status to evaluate the relationships between energy balance, ovarian reserve, and reproductive parameters more precisely. In addition, we used the dairy herd in which cows showed lower milk yield (6419 kg per 305 days in average) than average dairy cows in Japan (9601 kg per 305 days, 2016, Livestock Improvement Association of Japan, Inc.); therefore, we should examine the effects of the different ovarian reserves on reproductive parameters in higher yielding cows in future.

Previous studies reported that failed postpartum ovulation was due to an insufficient LH pulse frequency and lower estradiol production, and that NEB was a major factor responsible for this phenomenon [2, 7]. On the other hand, lower pulsatile LH secretion and higher sensitivity to LH in high AFC cattle were shown in recent studies; although high AFC cattle showed a lower LH pulse amplitude than low AFC cattle in the luteal phase [32], the circulating concentration of testosterone, which is produced in theca cells by an LH stimulus, was higher in high AFC cattle [33]. In addition, high AFC cattle showed higher intrafollicular androstenedione and estradiol concentrations in preovulatory follicles [33]. In a previous study, the effect of testosterone on the early follicular development was also reported; exogenous testosterone increased the number of secondary follicles [34]. These findings prompted us to speculate that the earlier first postpartum ovulation in high AFC cows in the present study was attributed to inherently higher sensitivity to LH than frequent LH pulses. In future studies, we need to investigate the frequency of LH pulses and expression of LH receptors in the dominant follicle in cows with different AFC.

The mean DIM of the first postpartum ovulation was previously reported to be 27.4 [35] and 30.9 [4]. Although the mean DIM of the first ovulation in all cows in the present study was 38.0 and later than those reported previously, mean DIM of the first ovulation was 26.6 in the high AFC group, which was consistent with previous findings. Previous studies reported that cows with an earlier first postpartum ovulation were subjected to significantly earlier first AI [36] and showed shorter days open [13, 36]. In contrast to these studies, the DIM of the first postpartum AI and mean days open were similar between the high and low AFC groups in the present study, in spite of the earlier first postpartum ovulation in high AFC cows. A reason for this may be that most cows (18 out of 23 cows) showed the first postpartum ovulation before VWP (60 DIM). In addition, the ovarian cycles of each cow were constantly examined by ultrasonography, and AI was performed regardless of the presence of obvious estrous behavior. Therefore, the frequent diagnosis of the ovarian status by ultrasonography may enhance the pregnancy of low AFC cows. Furthermore, higher ratio of multiparous cows conceived in high AFC group (6 out of 9, 66.7%) compared to low AFC group (2 out of 7, 28.6%), although days open were similar between high and low AFC groups. There is a possibility of high fertility especially in multiparous high AFC cows. In further study, we should examine the relationships between AFC, cow fertility, and the effect of frequent diagnosis by ultrasonography by the evaluation of more cows and different dairy herds.

Cows with a later first postpartum ovulation showed a shorter first ovarian cycle. This result was consistent with previous findings, which indicated that a short first ovarian cycle was frequently observed in dairy cows with the first ovulation later than 3 weeks postpartum [36, 37]. In the present study, cows with the first ovulation after 42 DIM (*n* = 5) belonged to the low AFC group and had a first ovarian cycle that was shorter than 12 days. These results suggest that low AFC cows have higher rates of a delayed first ovulation and short first ovarian cycle, leading to the delayed resumption of fertility, than high AFC cows. In a previous study on beef cows, a short first cycle was preceded by less frequent LH pulses and lower estradiol production during the first postpartum ovulatory growing phase [38]. The present results also indicate that low AFC cows have lower sensitivity to an LH stimulus and/or higher sensitivity to undernutrition condition than high AFC cows.

## Conclusions

The present study confirmed that first postpartum ovulation was earlier in cows with high AFC than in those with low AFC in undernutrition condition. Furthermore, cows with low AFC appear to be at a greater risk of delayed first postpartum ovulation and a short first ovarian cycle after the first postpartum ovulation, resulting in the delayed resumption of fertility, than cows with high AFC. However, frequent examinations of the ovarian cycles by ultrasonography may help earlier AI performance and pregnancy even in cows with delayed first postpartum ovulation. These results indicate that the ovarian reserve exerts strong regulatory effects on the timing of the first ovulation.

## Data Availability

The datasets used and/or analyzed during the present study are available from the corresponding author on reasonable request.

## Abbreviations

AFC: Antral follicle count
AI: Artificial insemination
AMH: Anti-Müllerian hormone
BCS: Body condition score
BW: Body weight
CL: Corpus luteum
DIM: Days in milk
FSH: Follicle-stimulating hormone
GnRH: Gonadotropin-releasing hormone
LH: Luteinizing hormone
NEB: Negative energy balance
VWP: Voluntary waiting period
